# The impact of recreational beach tennis associated with nutritional counseling on cardiometabolic risk factors in adolescents: a randomized clinical trial

**DOI:** 10.20945/2359-4292-2026-0124

**Published:** 2026-10-11

**Authors:** Dalva Muniz Pereira, Nathalia Jung, Rodrigo Pedroso, Gabriel Petry Heck, Luiz Felipe Holstein, Artur Pereira de Oliveira Irigoyen, Clair Rezende Fontana, Rodrigo Ferrari

**Affiliations:** 1 Programa de Pós-Graduação em Cardiologia, Faculdade de Medicina, Universidade Federal do Rio Grande do Sul, Porto Alegre, RS, Brasil; 2 Grupo de Estudos em Treinamento Físico e Esportivo, Centro de Pesquisa Clínica, Hospital de Clínicas de Porto Alegre, RS, Brasil; 3 Programa de Pós-Graduação em Ciências do Movimento Humano, Escola de Educação Física, Universidade Federal do Rio Grande do Sul, Porto Alegre, RS, Brasil

**Keywords:** Adolescence, exercise, nutrition guidelines

## Abstract

**Objective:**

To assess whether combining nutritional counseling with recreational sports confers greater improvements in cardiovascular risk factors among adolescents compared to recreational sports alone.

**Subjects and methods:**

This 12-week randomized clinical trial included 40 adolescents (aged 11-14 years; 67% female; 45% overweight/obese) randomly assigned to a beach tennis (BT, n = 20) or BT plus nutrition counseling (BT+N, n = 20) group. Sports sessions (BT) were performed twice a week and nutritional counseling (BT+N) once a week. Outcomes assessed at baseline and after 12 weeks included biochemical parameters (high- and low-density lipoprotein, total cholesterol; triglycerides; fasting glucose; and insulin), blood pressure, and body composition. Analyses were performed following the intention-to-treat principle.

**Results:**

Both groups showed significantly higher high-density lipoprotein levels (BT: +3.8 mg/dL, p < 0.05; BT+N: +5.9 mg/dL, p < 0.001) and no differences between groups. Fasting glucose decreased only in the BT+N group. Time x group interactions significantly differed between groups for triglycerides (p < 0.01), waist circumference (p < 0.001), body fat percentage (p < 0.05), and systolic blood pressure (p < 0.001), favoring the BT+N group. No significant changes were observed in low-density lipoprotein, total cholesterol, or insulin levels.

**Conclusion:**

Recreational BT alone improved some cardiometabolic parameters; however, its combination with nutritional counseling provided broader benefits, underscoring the value of integrated lifestyle interventions for reducing cardiometabolic risk factors in adolescents.

## INTRODUCTION

Adolescence is a critical period for developing habits that affect long-term health. Considering the prevalence of modifiable risk factors, preventive lifestyle measures are essential for reducing cardiovascular risks (^1^). Unhealthy eating habits, characterized by high energy density and low nutritional value, may directly influence blood pressure, insulin resistance (IR), and an atherogenic lipid profile through metabolic effects and by contributing to increased body weight and abdominal circumference (^2^). Physical activity confers numerous health benefits in adolescents, including improvements in body composition, cardiometabolic markers (e.g., lipid and glucose levels), reduced blood pressure, and positive effects on musculoskeletal and mental health (^3^). Despite these benefits, most adolescents, particularly females, do not meet recommended physical activity levels (^4^).

In developing countries, over half of all adolescents fail to achieve the recommended weekly physical activity volume for health, with inactivity being more prevalent among girls than boys (^5^). Regular sports engagement should be part of health promotion strategies for this age group, as it may reduce drop-out rates (^6^, ^7^). Beach tennis (BT), as a recreational and social sport, stands out for its rapid global expansion, ease of learning, and low motor complexity, making it accessible to different populations, including adolescents, and may promote adherence and conferring health benefits.

In addition to insufficient physical activity, many school-aged adolescents consume unhealthy diets characterized by low fruit and vegetable intake and frequent consumption of ultra-processed foods (^8^, ^9^). Establishing healthy dietary habits is essential for meeting adolescents' nutritional needs and may foster lifelong healthy behaviors (^10^).

Interventions that combine physical activity and nutritional counseling are effective in promoting health benefits in adolescents. Meta-analyses of randomized controlled trials indicate that combined dietary changes and physical activity lead to reductions in waist circumference (^11^), improvements in blood pressure (^12^), and enhancements in body composition (^13^). Nevertheless, few studies have explored a broad spectrum of cardiometabolic risk factors, and limited data are available on specific recreational sports, such as BT. Thus, a knowledge gap remains regarding the combined effects of recreational activities and dietary counseling on cardiometabolic outcomes in adolescents. This study aimed to investigate whether integrating nutritional counseling with recreational sports results in greater improvements in cardiometabolic riskfactors among adolescents than recreational sports alone.

## SUBJECTS AND METHODS

### Study design and participants

This parallel, randomized controlled clinical trial was conducted in the city of Porto Alegre (Rio Grande do Sul State, southern Brazil) between February and June 2025. Written informed consent was obtained from the participants' guardians, with their assent. The study was approved by the Ethics Committee of the Porto Alegre Clinical Hospital (approval no. 7.194.028) and registered in the Brazilian Registry of Clinical Trials (RBR-10bfs3gm). The trial is reported according to the Consolidated Standards of Reporting Trials (CONSORT) guidelines for randomized clinical trials (^14^).

Ninety-seven adolescents (aged 11-14 years of both sexes) were recruited from local public and private schools through school announcements and social media advertisements. Eligibility criteria included availability to participate per the predetermined schedule. Exclusion criteria were: medical conditions or injuries limiting protocol execution, medications affecting outcomes, pregnancy, or engagement in physical activity or nutritional counseling within the past six months. A sample size of 40 participants (20 per group) was determined to detect a minimum group difference of 8 mg/dL in high-density lipoprotein cholesterol (HDL-C) means (standard deviation = 8 mg/dL; a = 0.05; power = 80%), with an added 20% margin for possible losses and refusals. Full details of sample size calculation are available in the previously published protocol (^15^). **Figure 1** provides a flowchart of the study design.

**Figure 1:**
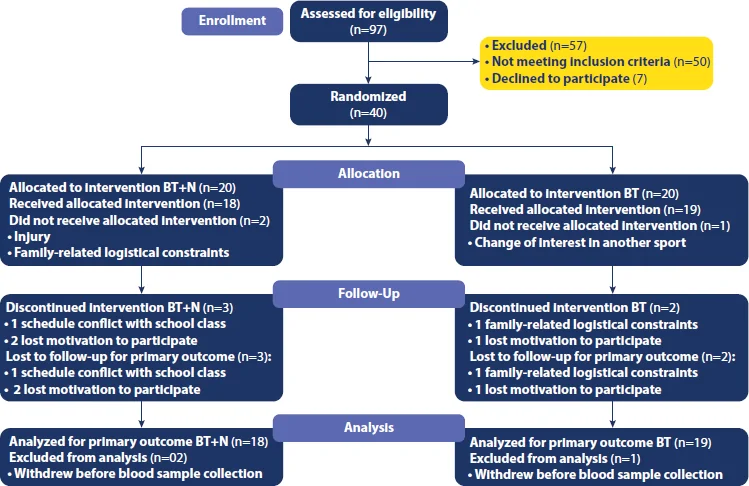
CONSORT flowchart of the study design.

#### Randomization

After stratification by sex and body mass index (BMI) status, participants were randomly assigned in a 1:1 ratio to either the BT or BT + nutrition counseling (BT+N) group by an independent researcher not involved in the study, following baseline assessment. Within each stratum, a simple randomization sequence was generated and automated using an artificial intelligence-based language tool.

#### Blinding

Due to the nature of the interventions, participants and intervention providers were not blinded to group Assignment. However, outcome assessors and data analysts remained blinded to group allocation throughout data collection and analysis.

#### Interventions

Both BT and BT+N interventions lasted 12 weeks. Beach tennis sessions occurred twice a week on non-consecutive days (60 min per session). In the BT+N group, nutritional counseling activities were done once a week (30 min per session). Interventions were conducted in a public university. Attendance was calculated as the ratio of sessions attended out of the total prescribed, for both BT and nutritional counseling. Intervention fidelity was ensured through standardized session protocols, attendance monitoring, and direct supervision by qualified staff. No modifications were made to the intervention or its delivery during the trial.

#### Recreational beach tennis

One week before the intervention, participants underwent two familiarization training sessions to learn BT fundamentals and assess skill level. This assessment helped to organize the matches with participants of similar proficiency levels in modality. Training sessions included: 1) warm-up exercises (strength and joint mobility), followed by specific low-intensity handball coordination drills; 2) technical-tactical exercises (racket-ball control, serve and return, drills performed solo and in pairs, with or without a net); 3) matchplay, comprising cooperative, mixed, and regulation matches (according to the official rules); 4) cool-down with coach feedback and sport-specific information. Water breaks were included as needed. Sessions took place twice a week (Monday/Wednesday or Tuesday/ Thursday) for 60 min each, in the afternoon (3:00-4:00 pm or 4:00-5:00 pm), scheduled to avoid conflict with school activities. Groups were organized according to participant availability, so not all participants trained simultaneously. Beach tennis periodization details are provided in **Figure 2.**

**Figure 2:**
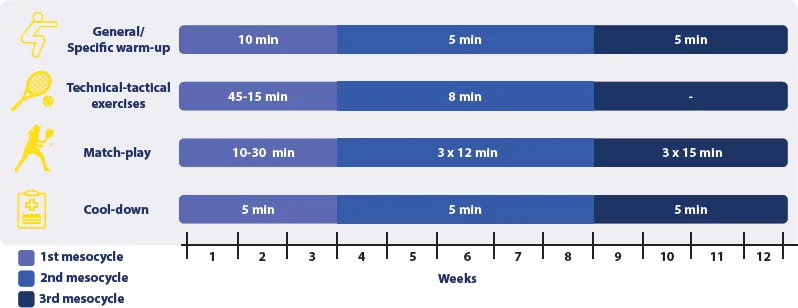
Structure and duration of the 12-week protocol.

#### Nutritional counseling

The nutritional intervention was coordinated by a registered dietitian and consisted of 12 educational sessions on healthy eating, based on the Brazilian Dietary Guidelines (^16^). Activities included discussions on food processing levels, reading food labels (e.g., fats, sugars, and sodium), interactive games, and practical strategies for improving diet quality. All activities were participatory and designed to promote engagement, learning, and healthier food choices. No participant was instructed to follow a pre-established diet or calorie restriction. Nutritional counseling occurred once a week, scheduled immediately after or before BT sessions to align with school schedules. At the end of the intervention, participants in the BT group received one individual nutritional counseling session. Adherence to both interventions was calculated as the number of sessions attended divided by the number of sessions offered.

#### Outcome measurements

All participants completed the same assessments at baseline (one week before the familiarization sessions) and after the 12-week intervention period. Assessments were conducted over multiple days, with participants evaluated in small groups during the morning to minimize circadian variation. Each assessment session lasted approximately 60 min per participant.

#### Biochemical parameters

Serum concentrations of HDL (primary outcome), low-density lipoprotein (LDL), total cholesterol, fasting glucose, fasting insulin, and triglycerides were determined at the Clinical Analysis Laboratory of the Hospital de Clínicas de Porto Alegre; IR was estimated using the homeostasis model assessment of IR (HOMA-IR).

#### Blood pressure

Systolic blood pressure (SBP) and diastolic blood pressure (DBP) were measured using a validated blood pressure monitor (HBP-1100, Omron Healthcare, Japan) with appropriately sized cuffs for arm circumference. Measurements were conducted according to the protocols of the Brazilian Guidelines of Hypertension (^17^).

#### Body composition

Body composition assessments were performed using a DEXA dual-energy X-ray absorptiometry (GE Lunar Healthcare®, USA) system with enCORE software, provided by the School of Physical Education, Physiotherapy, and Dance of the Federal University of Rio Grande do Sul. Lean mass, fat mass, and body fat percentage were quantified. During scanning, adolescents laid in the supine position on the examination table, with their lower limbs extended and slightly apart, arms positioned alongside their body, and thumbs facing upward. All metallic objects were removed. No participant experienced difficulty maintaining the required position throughout the assessment.

#### Anthropometric measurements

Body mass and height were measured using an analog scale with a stadiometer (Filizola, Brazil). Waist circumference was measured with an inextensible measuring tape (Sanny®, Brazil; precision 0.1 cm). Nutritional status was defined according to the BMI-for-age z-score, using World Health Organization cut-off points.

#### Food consumption

Food consumption was assessed through two individual, non-consecutive, 24-hour dietary recalls. Recalls were collected from the adolescent and analyzed using two Brazilian food composition tables (TACO and TBCA) (^18^, ^19^), which served as references for estimating mean intake of energy (kcal), carbohydrates (g), lipids (g), and proteins (g).

#### Responders to metabolic syndrome parameters

Participants were classified as “responders“ if they demonstrated a reduction of ≥5 cm in waist circumference (^20^); ≥5 mmHg in SBP (^21^); ≥10% reduction in triglycerides (^22^); ≥5 mg/dL increase in HDL-C (^23^); or ≥5 mg/dL reduction in fasting glucose (^24^).

### Statistical analysis

All analyses were conducted according to the intention-to-treat principle. The total sample included 40 participants, while specific analyses of biochemical parameters and body composition outcomes were based on the 37 participants who completed these assessments. Missing data were handled using multiple imputation. The assumption of normality was tested using the Shapiro-Wilk test. Quantitative variables are presented as means ± standard deviations with 95% confidence intervals (95% CI), while categorical variables are expressed as absolute and relative frequencies. For analysis of outcome variables, generalized estimating equations were applied, considering group (BT and BT+N), time (pre- and post-intervention), and group x time interactions. Changes (deltas) in variables were calculated, and groups were compared using analysis of covariance adjusted for baseline value and sex. Pairwise comparisons of estimated marginal means were adjusted for multiple testing using the Bonferroni correction.

We conducted a sub-analysis of 37 adolescents to investigate the proportion of responders for each component of metabolic syndrome. Outcomes were reported by variable and group, including the number and percentage of responders, risk ratio with 95% CI, and mean A (95% CI) from pre- to post-intervention. Risk ratios and their 95% CIs were estimated using Poisson regression models with robust variance. Differences in responder proportions between groups were compared using the *χ^2^* test. The analysis of improvement in the composite outcome (>3 improved components) was conducted using Poisson regression with robust variance, adjusted for sex, age, and BMI. All analyses were performed using SPSS software v. 19.0 (IBM Corp, USA). Statistical significance was set at *p* < 0.05.

## RESULTS

A total of 32 adolescents completed the intervention period. Of these, 45.2% were overweight or obese. Sociodemographic characteristics of the sample are described in **Table 1.** Adherence to the BT program was 85.8%, and adherence to nutritional counseling was 82.7%. During the intervention, two adolescents experienced minor adverse events during training sessions: one adolescent sustained an ankle contusion and another a wrist contusion. Both attended medical evaluations and missed two training sessions each, but subsequently completed the study without further issues.

**Table 1:** Sociodemographic and anthropometric characteristics of the sample at baseline

**Variable**	**BT(n** = 20)	BT + N(n = 20)	*p-* **Va lue**
Sex,n(%)			0.43
Male	07(35)	06(30)	
Female	13(65)	14(70)	
Age (years), means (SD)	13(0.9)	13(0.9)	0.27
Race/skin color, n (%)			0.25
White	18(90)	12(60)	
Black	01(5)	3(15)	
Mixed	01(5)	5(25)	
Anthropometric measurements, means (SD)			
Body mass, kg	58(16.4)	53(18.1)	0.09
Height, m	1.61(0.1)	1.55(0.1)	0.44
Waist circumference, cm	80(14.4)	78(14.7)	0.68
BMI-for-age, kg/m^2^	22(5.6)	22(6.5)	0.57
BMI percentile	67(29)	63 (33)	0.57
BMI z-score	0.7(1.2)	0.5(1.2)	0.55
School type, n (%)			0.82
Public	8(40)	7(35)	
Private	12(60)	13(65)	
Mother's level of education, n (%)			0.37
Incomplete elementary education	00(0)	01(5)	
High school education	06(30)	04(20)	
Incomplete undergraduate education	03(15)	04(20)	
Undergraduate education	11(55)	09 (45)	
Graduate education	00(0)	02(10)	
Household composition, n (%)			0.69
Both parents	11(55)	13(65)	
Only the mother	03(15)	01(5)	
Only the father	01(5)	01(5)	
Extended family	05(25)	05(25)	
Economic class (ABEP), n (%)		0.87
A	01(5)		04(20)
B	04(20)		07(35)
C	09 (45)		02(10)
D	04(20)		05(25)
E	01(5)		02(10)

ABEP: Brazilian Association of Research Companies; BMI: body mass index; BT: beach tennis group; BT+N: beach tennis + nutrition group; SD: standard deviation.

### Biochemical parameters

Both groups showed increased HDL levels, with no differences between groups. Triglycerides decreased only in the BT+N group, with a significant time x group interaction indicating a greater effect of the combined intervention **(Figure 3)**. Fasting glucose decreased only in the BT+N group, with no differences between groups over time. No significant changes were found for LDL, total cholesterol, insulin, or HOMA-IR **(Table 2).**

**Figure 3:**
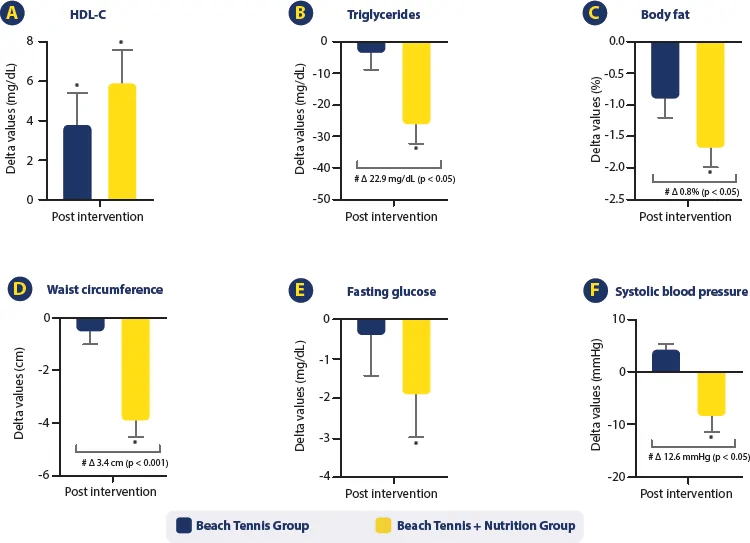
Mean A (pre-post change) after the 12-week protocol. *Within-group differences (p < 0.05); ^#^between group differences (*p* < 0.05).

**Table 2:** Body composition, biochemical parameters, and anthropometric measurements of the sample before and after the 12-week protocol

**Variable**	**BT(n** = 20)		**BT+N(n = 20)**		*p-* value*
	**Pre**	**Post**	**Pre**	**Post**	
Body composition					
Waist circumference, cm	79 (79-79)	78 (77-79)	80 (79-79)	75 (74-76)*	**&lt;0.001**
Body fat, %	34 (34-34)	34 (33-34)	34 (34-34)	33 (32-33)*	**&lt;0.05**
Fat mass, kg	20 (20-20)	20(19-20)	20 (20-20)	20(19-20)	0.56
Lean mass, kg	35 (35-35)	36 (36-37)	35 (35-35)	37 (36-37)	0.41
Hemodynamic parameters					
SBP, mmHg	103(102-105)	107(103-110)	103(102-104)	94(92-96)*	**&lt;0.001**
DBP, mmHg	57 (56-58)	59(57-61)	56 (55-57)	58 (56-60)	0.12
Heart rate, bpm	82(81-83)	85 (82-88)	82(81-83)	85(81-89)	0.79
Lipid parameters					
HDL-C, mg/dL	42 (42-43)	46 (43-50)*	42 (42-43)	48(46-51)*	0.37
LDL-C, mg/dL	87 (86-88)	89 (84-94)	89(87-91)	85(81-90)	0.59
Total cholesterol, mg/dL	144(143-146)	150(143-157)	148(145-150)	146(140-152)	0.85
Triglycerides, mg/dL	71 (68-75)	70 (58-82)	73 (69-76)	46 (36-56)*	**&lt;0.01**
Glycemic parameters					
Fasting glucose, mg/dL	85 (84-86)	84 (82-86)	85 (85-86)	82(81-84)*	0.32
Fasting insulin, uU/mL	2.2(2.0-2.3)	2.2(2.0-2.5)	2.1 (2.0-2.3)	2.1 (1.9-2.3)	0.17
HOMA-IR	0.6(0.5-0.6)	0.6(0.5-0.8)	0.6 (0.5-0.6)	0.5(0.3-0.7)	0.15

BT: Beach tennis group; BT+N: beach tennis + nutrition group; DBP: diastolic blood pressure; HDL-C: high-density lipoprotein cholesterol; HOMA-IR: homeostasis model assessment for insulin resistance (glucose [mg/dL] x insulin [uU/mL])/40; LDL-C: low-density lipoprotein cholesterol; SBP: systolic blood pressure.*Significant difference compared with baseline within the same group (*p* &lt;0.05); values are provided as means (95% confidence interval); ^#^values refer to time x group interactions.

### Body composition and anthropometric measurements

After 12 weeks, significant time x group interactions were observed for waist circumference and body fat percentage, with reductions exclusively in the BT+N group and no changes in the BT group **(Figure 3)**. Body weight, BMI, and lean mass increased in both groups, with no between-group differences. No significant changes were found for fat mass **(Table 2).**

### Blood pressure

A significant between-group difference was observed for SBP change at the end of the intervention, with a reduction in the BT+N group and no significant change in the BT group **(Figure 3**). No differences were found between groups for DBP or heart rate after the intervention **(Table 2).**

### Food consumption

Total energy intake did not change significantly over time in the BT group (Δ = 13.3 kcal; *p* = 0.45) or in the BT+N group (Δ = -37.8 kcal; *p* = 0.14). The BT+N group exhibited a significant reduction in carbohydrate intake (Δ = -8.1 g; *p* < 0.05), whereas no significant change was observed in the BT group (Δ = 0.7 g; *p* = 0.59). The time x group interaction was significant (*p* < 0.05), indicating a greater reduction in carbohydrate intake with the combined intervention. Protein intake increased significantly in the BT+N group (Δ = 2.1 g; *p* < 0.05), while the BT group showed a nonsignificant increase (Δ = 2.9 g; *p* = 0.08). Lipid intake remained stable in both groups: BT group (Δ = -0.15 g; *p* = 0.66); BT+N group (Δ = -1.6 g; *p* = 0.07). No statistically significant differences were observed between groups for protein (*p* = 0.47) or lipid (*p* = 0.07) intake.

### Responders to metabolic syndrome parameters

The combined intervention (BT+N) resulted in a higher proportion of responders for SBP and triglycerides, with statistically significant differences (*p* < 0.01). The BT+N group also showed a higher proportion of responders for waist circumference (33.3% vs. 5.3% in BT), with a marginal, non-significant difference (*p* = 0.07) **(Table 3).** In the composite outcome analysis, 83.3% of participants in the BT+N group showed improvements in three or more metabolic syndrome parameters, whereas only 15.8% of participants in the BT group achieved the same result.

**Table 3:** Analyses of responders for components of metabolic syndrome and estimated risk ratio of the sample after the 12-week protocol

**Variable**	**BT**	**BT + N**	*p*(*x*^2^)	**Risk ratio (95% Cl)**	***p* (Poisson)**
	**n** (%)				
WC ↓ ≥5 cm	1 (5.3)	6(33.3)	**&lt;0.05**	6.33 (0.84-47.6)	0.07
SBP ↓ ≥5 mmHg	4(21.1)	14(77.8)	**&lt;0.01**	3.69(1.49-9.13)	**&lt;0.01**
TG ↓≥10%	8(42.1)	17(94.4)	**&lt;0.01**	2.24(1.31-3.85)	**&lt;0.01**
HDL-C ↑ ≥5mg/dL	11(57.9)	-15(83.3)	0.09	1.44(0.93-2.23)	0.10
Glucose ↓ ≥5 mg/dL	5(26.3)	6(33.3)	0.64	1.27(0.47-3.43)	0.64

BT: Beach tennis group; BT+N: beach tennis + nutrition group; 95% CI: 95% confidence interval; HDL-C: high-density lipoprotein cholesterol; SBP systolic blood pressure; TG triglycerides; WC: waist circumference; ?2: Chi-square test.

## DISCUSSION

Recreational BT, alone or combined with nutritional intervention, promoted increases in HDL-C, highlighting the importance of early interventions to improve lipid profiles and prevent cardiovascular diseases. However, only the combined intervention resulted in significant reductions in waist circumference, body fat percentage, triglyceride levels, and SBP. Consistent with our findings, a meta-analysis of combined interventions in adolescents demonstrated superior effectiveness versus exercise or diet alone in reducing BMI, body fat, and waist circumference, as well as lowering triglycerides and LDL cholesterol (^25^). These results suggest that the addition of nutritional guidance enhances the positive effects of physical activity, reinforcing the importance of multicomponent strategies for preventing and managing cardiometabolic risk factors in adolescents.

Abdominal obesity is a key cardiometabolic risk factor in adolescents. The ERICA study, which included 37,504 Brazilian adolescents aged 12-17 years, identified elevated waist circumference as a central component of metabolic syndrome according to the International Diabetes Federation criteria. The most frequent component combinations, accounting for three-quarters of cases, were high waist circumference, low HDL-C, and high blood pressure; high waist circumference, low HDL-C and high triglycerides; and high waist circumference, low HDL, high triacylglycerols, and high blood pressure (^26^). Demonstrating the modifiable potential of this indicator, a study in India with overweight adolescents showed that after nine months of a nutritional education program with sessions on healthy eating, waist circumference significantly decreased in the intervention group, while the control group maintained stable values (^27^).

The significant reduction in body fat percentage in the BT+N group further suggests that BMI alone may underestimate changes in body composition during adolescence, due to linear growth and possible gains in lean mass associated with exercise. Variation in fat percentage provides a more robust description of intervention effects (^28^). A 16-week multidisciplinary intervention in children and adolescents, combining nutritional guidance, lifestyle counseling, psychological support, and thrice-weekly exercise, produced significant group-by-time interaction effects on body fat percentage (^29^). In addition, observational studies in children and adolescents have indicated a positive association between higher added sugar intake and elevated triglyceride levels (^30^, ^31^). In this context, nutritional interventions promoting the reduction of sweets, sugar-sweetened beverages, and other sources of simple sugars can improve lipid profiles, including triglyceride reduction, as observed in the combined intervention group in this study.

Most recreational sports, including BT, are characterized by intermittent modalities, alternating low-to-moderate intensity with high-intensity bouts, since the game involves constant forward and backward movement, jumps, and upper body activity. These characteristics are comparable to traditional aerobic interval exercise protocols, resulting in significant cardiovascular demand during matches (^32^). The increased energy cost of movement on sand surfaces (e.g., BT courts) enhances overall caloric expenditure and lipid oxidation (^33^), making this modality an effective exercise strategy for improving body composition.

Elevated blood pressure during childhood or adolescence is associated with various intermediate markers and adverse cardiovascular outcomes in adulthood (^34^, ^35^). Nutritional interventions emphasizing healthy eating have demonstrated significant effects on blood pressure in adolescents (^36^), as both consumption of sodium-rich foods (^37^) and excessive intake of carbohydrates and fats are linked to hypertension in this population (^38^). Non-pharmacological strategies have proven effective in reducing blood pressure in healthy youth, as shown in a network meta-analysis of children and adolescents, which revealed that interventions combining physical activity, diet, and behavioral modification were more effective at lowering both SBP and DBP (^12^). Hence, the observed reduction in SBP may be explained by the synergistic effects of a healthy diet and physical exercise, likely resulting in more effective blood pressure modulation than BT alone.

Despite the positive effects of the interventions on several health-related outcomes, BMI, fat mass, DBP, and certain biochemical parameters (e.g., LDL, total cholesterol, glucose, insulin, and HOMA-IR) did not change significantly throughout the intervention period. This may be partially attributed to the intervention duration, as more stable metabolic outcomes (e.g., lipid profiles and IR) generally require longer periods to demonstrate statistically significant changes in adolescents (^39^). Additionally, many baseline values were within normative ranges and did not exceed the clinical cut-off points for metabolic risk, potentially limiting the scope for observable reductions. This also reflects the relatively healthy metabolic profile of the participants, as this study aimed to assess the preventive effects of lifestyle interventions in a general adolescent population, rather than in a clinical sample with metabolic disorders.

This study has some potential limitations. The analyzed indicators were restricted to selected parameters and did not encompass other potential benefits. Additionally, the intervention duration may have been insufficient to detect significant changes in more stable outcomes. A further limitation is that most participants presented metabolic parameters within normal ranges at baseline, which may have limited measurable improvements in cardiometabolic markers. Nevertheless, this randomized controlled trial has important strengths. The intervention was innovative, combining recreational BT with structured nutritional counseling and offering a multicomponent strategy to promote cardiometabolic health in this population. Adherence to both exercise and counseling sessions was high, supporting the feasibility and acceptability of the program.

In conclusion, sports participation during adolescence is associated with a wide range of physical, psychological, and social benefits (^6^). Beach tennis is a recreational activity notable for its social nature, typically played in pairs, fostering social interaction. The additional improvements observed in the group receiving the combined intervention suggest that nutritional counseling potentiated the effects of physical exercise, reinforcing the importance of early lifestyle interventions that integrate both exercise and nutrition for the prevention of future cardiovascular risk. To our knowledge, this is the first randomized controlled trial to demonstrate the benefits of a rapidly growing recreational sport in adolescents, further supporting the understand that integrating physical activity programs with nutritional education aimed at the primary prevention of cardiovascular diseases in youth represents a promising and feasible public health strategy.

## Data Availability

datasets related to this article will be available upon request to the corresponding author.
